# Lemierre's Syndrome: A Case Report

**DOI:** 10.7759/cureus.34473

**Published:** 2023-01-31

**Authors:** Leticia Santos, Filipa Monteiro, Maria Marques, Rita Homem, Vanda Spencer

**Affiliations:** 1 Internal Medicine, Hospital Garcia de Orta, Almada, PRT

**Keywords:** jugular vein thrombophlebitis, septic embolism, septic thrombophlebitis, fusobacterium necrophorum, lemièrre’s syndrome

## Abstract

Lemierre's syndrome is a rare condition characterized by septic thrombophlebitis of the internal jugular vein secondary to infection of the head and neck region and septic embolization to other organs. The most frequent etiological agent is *Fusobacterium necrophorum*, a commensal anaerobic gram-negative bacillus of the oral flora. We report the case of a young male who presented with chest pain after a dental procedure. He developed a masseterian phlegmon, thrombosis of the internal jugular vein, and embolization to the lung complicated by empyema. The diagnosis of Lemierre's syndrome was delayed by the negative blood cultures, but full recovery was achieved after appropriate broad-spectrum antibiotic coverage. Our main objective is to highlight the fact that a high clinical suspicion is required to establish the diagnosis of this rare syndrome.

## Introduction

Lemierre’s syndrome or post-anginal sepsis, as described by Andre Lemierre in a study of 20 cases in 1936, is a rare condition with an estimated incidence of 1/1.000.000 per year and typically affects teenagers and young adults [1,2,3]. It is characterized by septic thrombophlebitis of the internal jugular vein secondary to an infection of the head and neck region caused by anaerobic septic embolization [2]. The most common etiological agent is *Fusobacterium necrophorum*, an anaerobic commensal gram-negative bacillus of the oral flora; however, it may also be caused by other agents such as *Staphylococcus, Streptococcus, Proteus,* and* Bacteroides* [3].

## Case presentation

The patient was a healthy 31-year-old male patient with no toxicophilic habits, who was admitted to the Emergency Department (ED) for pleuritic right thoracic pain three days after a dental extraction. Prior to the dental extraction, the patient had been medicated with amoxicillin and clavulanic acid for seven days due to a dental abscess. The patient denied other complaints, including dyspnea, cough, fever, and gastrointestinal or urinary symptoms.

At observation in the ED, he presented with fever (tympanic temperature of 39 ºC) and tachycardia (heart rate of 131 bpm); pulmonary auscultation showed decreased right vesicular murmur, cardiac auscultation with rhythmic sounds without murmurs and abdominal observation revealed tenderness on the right hypochondrium. Complete blood count (Table 1) showed leukopenia (1900/µL, with 87% neutrophilia), elevated C-reactive protein (31.5 mg/dL), and elevated total bilirubin (2 mg/dL), and hyperlactacidemia (of 3 mmol/L) was found in arterial blood gas analysis.

**Table 1:** Laboratory results at the time of admission

Test	Result (on admission)	Reference range
Hemoglobin	146 g/L	115–180 g/L
White blood cells	1.9 x 10^9^/L	4.0–11.0 x 10^9^/L
Neutrophils	1.65 x 10^9^/L	1.9–8.0 x 10^9^/L
Lymphocytes	0.22 x 10^9^/L	0.9–5.2 x 10^9^/L
Platelets	174 x 10^9^/L	130–400 x 10^9^/L
Prothrombin time	12.6 seconds	9.4–12.5 seconds
Partial thromboplastin time	35.2 seconds	25.1–36.5 seconds
D-dimer	1.10 ug/mL	<0.23 ug/mL
Sodium	140 mmol/L	135–145 mmol/L
Potassium	3.8 mmol/L	3.5–5.0 mmol/L
Blood urea nitrogen	25 mg/dL	16–48 mg/dL
Creatinine	1.1 mg/dL	0.7–1.2 mg/dl
Aspartate aminotransferase	39 UI/L	<40 UI/L
Alanine aminotransferase	90 UI/L	<50 UI/L
Lactate dehydrogenase	307 UI/L	240–480 UI/L
Direct bilirubin	0.97 mg/dL	<0.3 mg/dL
Total bilirubin	2 mg/dL	<1.2 mg/dL
Troponin T hs	<13 ng/L	<14 ng/L
C-reactive protein	31.5 mg/dL	<0.2 mg/dL
Procalcitonin	0.83 ng/mL	<0.5 ng/ml

**Figure 1:**
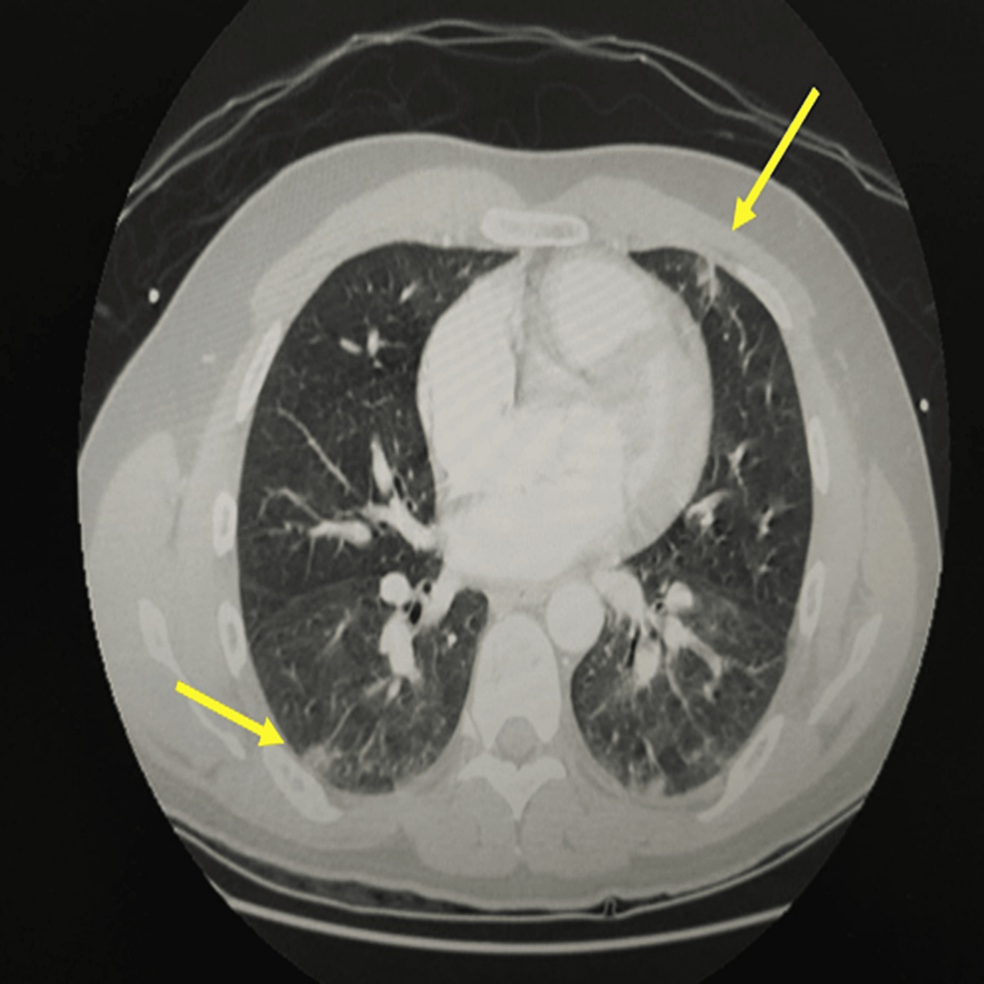
Thoracoabdominal CT showing pulmonary nodular lesions with central cavitation suggestive of septic embolization CT: computed tomography

**Figure 2:**
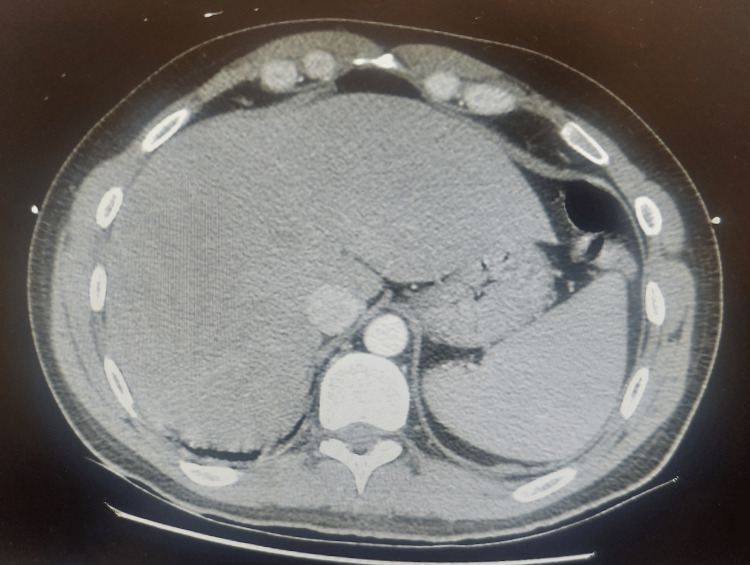
Abdominal CT showing hepatosplenomegaly CT: computed tomography

The patient underwent thoraco and abdominal CT (Figures 1, 2), which revealed pulmonary nodular lesions with central cavitation suggestive of septic embolization as well as hepatosplenomegaly.

The hypothesis of endocarditis with pulmonary embolism after a dental procedure was considered and the patient was admitted to the Intermediate Care Unit. After blood samples were drawn for microbiological analysis, empirical antibiotic therapy with ceftriaxone, vancomycin, and gentamicin was started. The bedside transthoracic echocardiogram showed no images suggestive of vegetation. Given his clinical stability, the patient was transferred to the Internal Medicine ward on the third day of hospitalization. The transesophageal echocardiography excluded the hypothesis of endocarditis and the antibiotic therapy was de-escalated to ceftriaxone and clindamycin.

**Figure 3:**
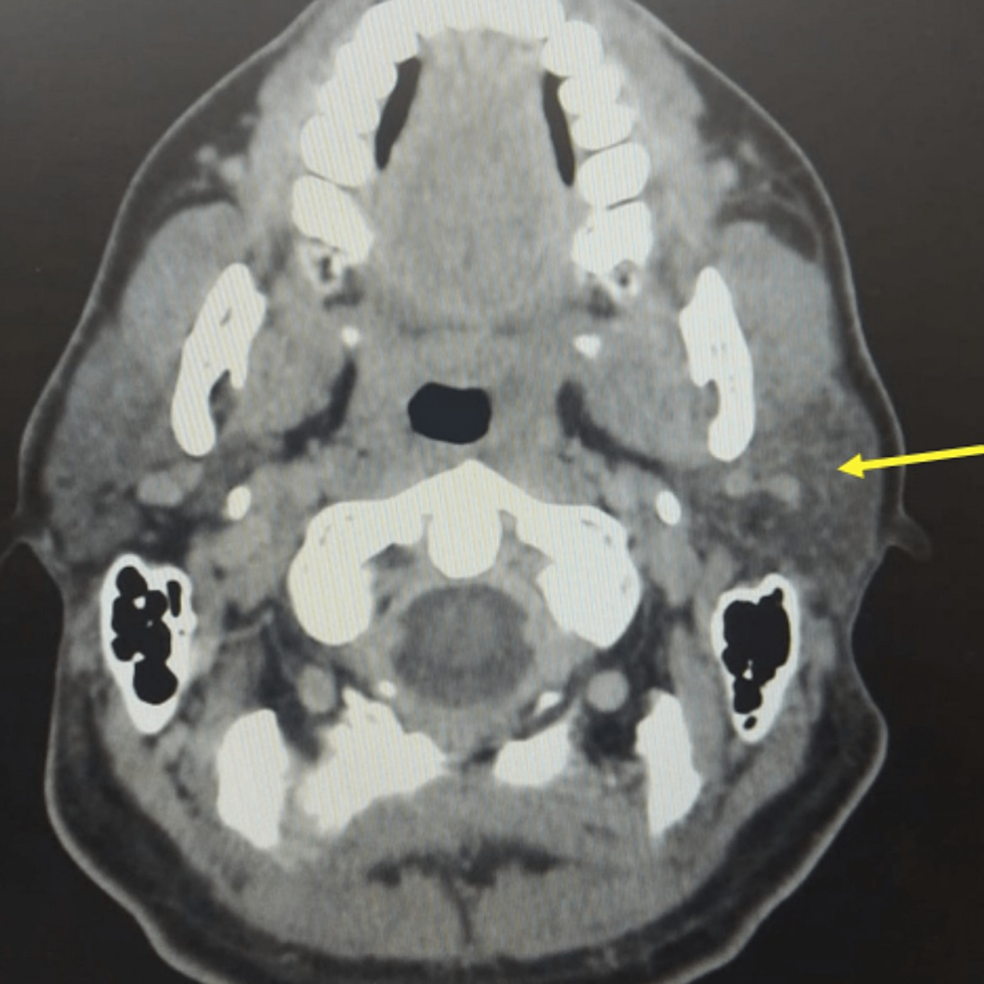
Facial CT revealing an abscess adjacent to the left ascending branch of the mandible, within the masseter CT: computed tomography

**Figure 4:**
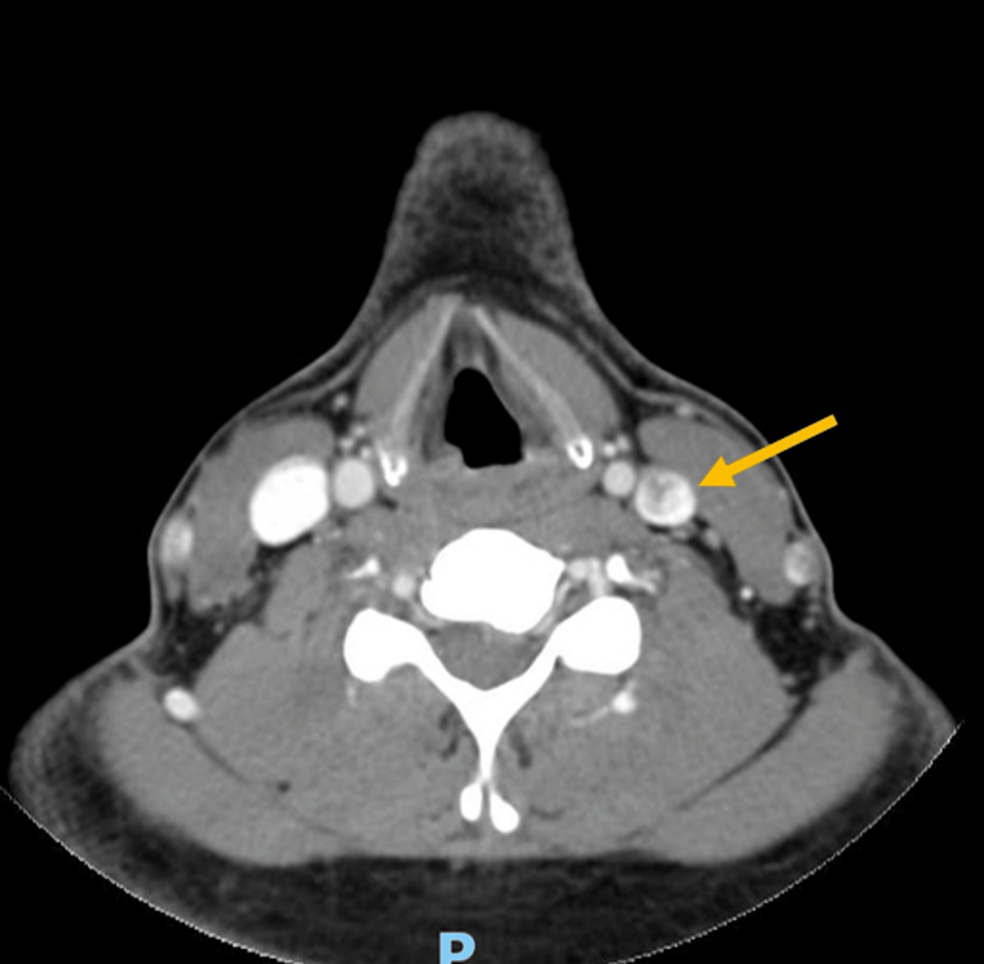
Facial CT showing a heterogeneous opacification of the jugular vein compatible with partial thrombosis CT: computed tomography

During hospitalization, the patient was evaluated by the maxillofacial surgery team and no purulent discharge was detected on the extraction site as well as edema of the neck or oropharynx. A facial CT scan, facial bone scintigraphy, and tympanogram were performed for eventual referral to Hyperbaric Oxygen Therapy as adjuvant therapy. The facial CT showed a missing dental piece 3.6 and a cyst in this region with gaseous focus and continuity solution for the adjacent thickened soft spaces, suggestive of phlegmon. An abscess adjacent to the left ascending branch of the mandible, within the masseter, and ganglion asymmetry with enlargement of the left lymph nodes were observed (Figure 3). This confirmed the origin of the infection and the scintigraphy showed similar results. A heterogeneous opacification of the jugular vein compatible with partial thrombosis was also observed in the facial CT scan (Figure 4), which was confirmed by a venous Doppler ultrasound. 

**Figure 5:**
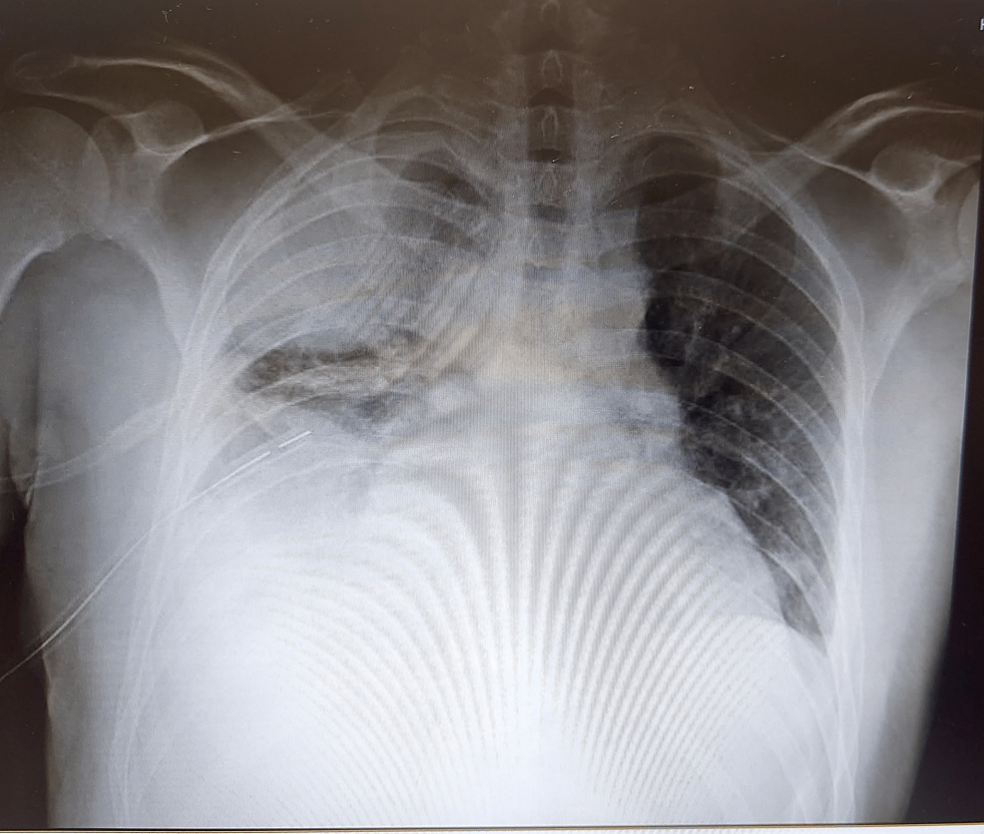
Chest X-ray showing a right pleural effusion

**Figure 6:**
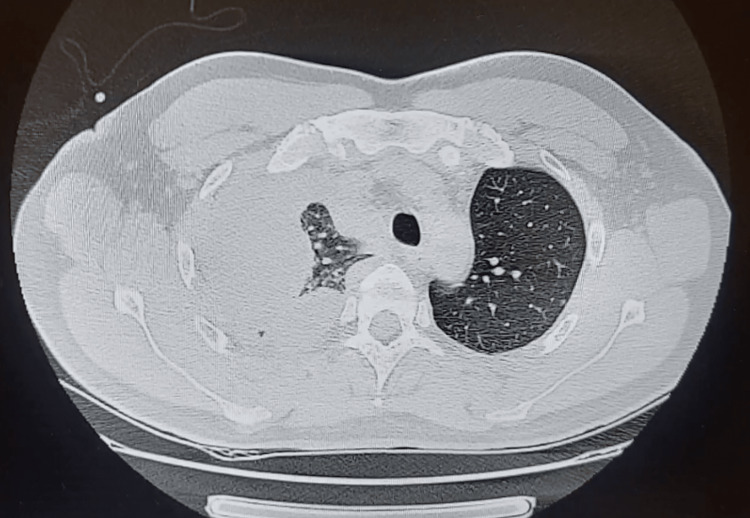
Thoracoabdominal CT showing right massive pleural effusion with mild pleural thickening CT: computed tomography

After initial clinical improvement, the patient showed abrupt clinical worsening with severe dyspnea, recurrence of fever, and increased C-reactive protein and procalcitonin. Chest X-ray (Figure 5) showed pleural effusion, which was confirmed by a CT scan (Figure 6), showing a right massive pleural effusion with mild pleural thickening and almost complete collapse of the ipsilateral lower and middle lobes. The pleural effusion was drained and was suggestive of empyema (pH 7.11). New blood cultures were performed.

The patient underwent pleural debridement and lobar decortication and curettage of the tooth extraction site. Given the presence of a dental abscess, internal jugular vein thrombosis, and pulmonary septic embolization, Lemierre’s syndrome was considered. The patient completed six weeks of antibiotic therapy with clinical and radiological improvement. The Doppler ultrasound performed before hospital discharge showed permeability of the visible sectors of the internal and external jugular veins and the chest CT showed improvement in the pleuroparenchymal findings. All five blood cultures collected grew no organisms. The patient achieved full recovery and was asymptomatic on discharge as well as on the follow-up appointments at one, six, and 12 months after discharge.

## Discussion

The most common etiological agent for Lemierre's syndrome is *Fusobacterium necrophorum*, an anaerobic commensal gram-negative bacillus of the oral flora; however, it may also be caused by other agents such as *Staphylococcus, Streptococcus, Proteus, and Bacteroides* [3]. Hematogenous dissemination results in sepsis and distant septic embolization, predominantly to the lung, which may progress to abscesses and empyema in 10-15% of cases [1,2,4,5,6]. Fever, chills, and other accompanying symptoms usually recur within about a week after the initial oropharyngeal infection, by which time it may have resolved or ameliorated [1,4,5]. For the diagnosis, besides clinical presentation, cultural specimens (which may take a few days) and imaging exams such as echo-Doppler and CT are essential [2].

Treatment consists of prolonged anaerobic-coverage antibiotic therapy (three to six weeks) and surgical drainage of abscesses or debridement of necrotic tissue, if necessary [2,5]. Therapeutic anticoagulation to expedite the resolution of the septic thrombus and prevent its spread (especially to the central nervous system) and embolization remains controversial and no clear recommendations exist regarding its use [6]. Most authors agree that it should be reserved for high-risk patients [2,6]. The mortality rate varies from 5 to 25% in the literature (up to 90% in the pre-antibiotic era), and it is well known that any delay in the diagnosis and treatment increases the morbidity and mortality associated with this illness [2,3,6]. 

Due to its rarity, a high clinical suspicion is required to establish the diagnosis of Lemierre's syndrome. In our case, the diagnosis was achieved later in the course of the hospitalization after the assembly of the recent dental infection, the radiological evidence of internal jugular vein thrombosis, and septic embolization to the lung (the most commonly affected organ). No agent was identified in the blood cultures, possibly suppressed by the prior administration of antibiotics, which deviates from the classical presentation of this syndrome. Even after clinical worsening, ceftriaxone and clindamycin were maintained assuming it did not occur in the context of antibiotic therapy failure but rather the absence of focus control, which was later performed. Therapeutic anticoagulation was not performed, due to the risks and the lack of clear benefits described in the literature. A full clinical recovery was ultimately achieved.

## Conclusions

We presented a case of a young male admitted with chest pain after a dental infection. Although there was no evidence of bacteriemia in the blood cultures, thrombosis of the internal jugular vein and embolization to the lung further complicated by empyema support the diagnosis of Lemierre's syndrome. Empirical antibiotic therapy was started and resulted in the full recovery of the patient.

We intend to increase awareness among physicians about this rare condition, for which a high suspicion is needed to achieve the diagnosis, which may be delayed due to the range of clinical findings of septic embolization. Lemierre's syndrome usually has a good prognosis after proper and timely treatment, which is critical since it can be otherwise life-threatening due to embolic complications. Until further evidence becomes available, antibiotic therapy remains the basis of treatment, with surgical drainage if required and anticoagulation in selected cases.
